# Impact of preovulatory follicle maturity on oocyte metabolism and embryo development

**DOI:** 10.1093/pnasnexus/pgae181

**Published:** 2024-04-30

**Authors:** Hannah M Clark, Allyson E Stokes, J Lannett Edwards, Rebecca R Payton, F Neal Schrick, Shawn R Campagna, Qudus Sarumi, Emma A Hessock, Samantha R Roberts, Nima Azaridolatabad, Sarah E Moorey

**Affiliations:** Department of Animal Science, University of Tennessee Institute of Agriculture and AgResearch, 2506 River Drive, Knoxville, TN 37996, USA; Department of Animal Science, University of Tennessee Institute of Agriculture and AgResearch, 2506 River Drive, Knoxville, TN 37996, USA; Department of Animal Science, University of Tennessee Institute of Agriculture and AgResearch, 2506 River Drive, Knoxville, TN 37996, USA; Department of Animal Science, University of Tennessee Institute of Agriculture and AgResearch, 2506 River Drive, Knoxville, TN 37996, USA; Department of Animal Science, University of Tennessee Institute of Agriculture and AgResearch, 2506 River Drive, Knoxville, TN 37996, USA; Department of Chemistry, University of Tennessee, 1420 Circle Dr., Knoxville, TN 37996, USA; Department of Chemistry, University of Tennessee, 1420 Circle Dr., Knoxville, TN 37996, USA; Department of Animal Science, University of Tennessee Institute of Agriculture and AgResearch, 2506 River Drive, Knoxville, TN 37996, USA; Department of Animal Science, University of Tennessee Institute of Agriculture and AgResearch, 2506 River Drive, Knoxville, TN 37996, USA; Department of Animal Science, University of Tennessee Institute of Agriculture and AgResearch, 2506 River Drive, Knoxville, TN 37996, USA; Department of Animal Science, University of Tennessee Institute of Agriculture and AgResearch, 2506 River Drive, Knoxville, TN 37996, USA

**Keywords:** bovine, cumulus-oocyte complex metabolism, embryo development, follicular fluid, preovulatory follicle

## Abstract

Improved oocyte competence for embryo development and pregnancy was observed following ovulation of preovulatory follicles with greater physiological maturity, as indicated by estradiol production, prior to the gonadotropin-releasing hormone (GnRH)-induced luteinizing hormone (LH) surge. It was hypothesized that follicular fluid from preovulatory follicles of greater maturity better supports the maturing oocyte's metabolic requirements and improves embryo development. The objective was to determine if differences in preovulatory follicular fluid due to follicle maturity influence oocyte metabolism during in vitro maturation (IVM) and affect embryo development. Bovine preovulatory follicular fluid was collected 18 h after a GnRH-induced LH surge. Serum estradiol concentration at GnRH administration categorized follicles as greater or lesser maturity. Immature bovine oocytes were submitted to 24 h IVM in medium supplemented with 20% follicular fluid from preovulatory follicles of greater or lesser maturity. Embryo development was recorded. Oocyte maturation media and media conditioned by developing embryos were submitted for metabolomics. A randomized block design was utilized to determine differences in embryo development and media metabolites (*P* ≤ 0.05). Blastocysts from oocytes matured in greater vs. lesser maturity follicular fluid had a more moderate rate of development (*P* = 0.01). At the conclusion of 24 h IVM, abundance of 66 metabolites differed between greater and lesser follicle maturity treatments. Nine metabolites differed in media conditioned by developing embryos. Metabolome results suggest improved amino acid, purine, and glucose metabolism, followed by a more efficient rate of embryo development, in oocytes matured in greater vs lesser maturity follicular fluid.

**Significance Statement**

The oocyte's environment during maturation affects its competence for embryo development and pregnancy. Previous studies identified a relationship between bovine preovulatory follicle maturity and the follicular fluid metabolome, oocyte competency for embryo development, and pregnancy success. Results indicate that follicular fluid from preovulatory follicles of greater vs lesser maturity modified oocyte metabolism to promote a more efficient rate of embryo development. Findings demonstrate causality between follicular fluid milieu from greater or lesser mature preovulatory follicles and oocyte metabolic preparation for embryo development. Specific modifications in metabolite abundance following in vitro maturation provide targets for improving oocyte maturation environment to increase reproductive efficiency in cattle that may be relevant for other agriculturally relevant species and humans.

## Introduction

Improving fertility in both humans and domestic livestock has been a high priority for decades, prompting the development of multiple assisted reproductive technologies. Numerous factors influence fertility, but production of a mature oocyte with competency to develop into a quality embryo is essential for pregnancy to occur. The in vivo maturing oocyte is enclosed within a preovulatory follicle, and in vivo maturation of bovine oocytes led to blastocyst development and hatching that were two times higher than those matured in vitro (1). Requirement of oocyte maturation within the preovulatory follicle for optimal embryo development is not surprising, as the preovulatory follicular fluid milieu plays an important role as the oocyte undergoes modifications that promote its acquisition of developmental competence (2, 3). Substrates produced by the granulosa cells are released into the follicular fluid that bathes the cumulus-oocyte complex (COC) and provides the maturing oocyte with an environment supportive of oxidative balance and carbon metabolism for energy, amino acid synthesis, and protein production (4–7). The preovulatory follicular fluid's unique capacity to support the maturing oocyte is further observed through distinct modifications to the preovulatory follicular fluid metabolome during progression of bovine oocyte maturation. Follicular fluid abundance of metabolites critical for glucose and amino acid metabolism were increased at approximately 11 and 18 h after the luteinizing hormone (LH) surge compared to before the LH surge (8).

Production of an optimal intrafollicular environment is essential to support the oocyte as it matures and prepares for ovulation, fertilization, early embryo development, and pregnancy. In the days before the LH surge, the preovulatory follicle undergoes a period of increased growth and estradiol production as the follicle advances in maturity (9). The preovulatory LH surge instigates follicular cell differentiation, with the follicular fluid environment ever evolving as the oocyte matures within (10). Physiological maturity of the preovulatory follicle, indicating its readiness nurture the maturing oocyte, is positively associated with circulating estradiol concentration and follicle size (11). In cattle (11–13), humans (14), and swine (15, 16) the maturity of the preovulatory follicle at the time of a gonadotropin releasing hormone (GnRH)-induced LH surge affects establishment and maintenance of pregnancy. Increased preovulatory follicle diameter and higher levels of circulating estradiol at GnRH administration were associated with improved embryo quality and increased overall embryo cleavage and blastocyst development rates (12, 13). Interestingly, preovulatory follicle diameter was also related to a more moderate rate of in vivo embryo development (12).

The relationship of preovulatory follicle maturity, based on follicle size and estradiol production, with oocyte developmental competence appears to be at least partially metabolic in nature. Abundance of intraoocyte adenosine triphosphate, oocyte transcripts essential for oxidative phosphorylation, and cumulus cell transcripts essential for glucose metabolism were positively related to greater maturity of the preovulatory follicle at the GnRH-induced LH surge in bovine (17, 18). Improved follicular fluid capacity to support metabolic requirements of the COC was also observed in bovine preovulatory follicles of greater maturity immediately before the GnRH-induced LH surge because the abundance of numerous follicular fluid metabolites critical for energy and protein production was higher in follicles of larger size or higher estradiol production (18, 19).

The evidence above suggests that preovulatory follicles of greater physiological maturity prior to the LH surge more readily provide substrates required for cumulus cell and oocyte metabolism which may directly influence oocyte competence for embryo development. Therefore, it was hypothesized that follicular fluid originating from preovulatory follicles of greater physiological maturity prior to the GnRH-induced LH surge would better support metabolic requirements of the maturing oocyte and metabolically program the oocyte to affect embryo development. The objective of this study was to determine if differences in preovulatory follicular fluid due to follicle physiological maturity directly influence oocyte metabolism during in vitro maturation (IVM) and affect subsequent embryo development.

## Results

### Embryo development after COC exposure to preovulatory follicular fluid supplementation 24 h IVM

Bovine COCs were matured in oocyte maturation media (OMM) supplemented with 20% preovulatory follicular fluid of cows in the upper third (greater follicle maturity, 11.8 ± 0.37 pg/mL) or lower third (lesser follicle maturity, 3.7 ± 0.32 pg/mL) for serum estradiol concentration prior to the GnRH-induced LH surge. Distinction between follicle classifications was pronounced, as serum estradiol concentration was >2 fold higher in all animals of greater vs lesser follicle maturity. Total number of COCs (*n* = 1815), along with their relevant developmental progression after performing in vitro fertilization (IVF) are depicted in Table 1. The percentage of putative zygotes recovered or lysed 16 h post-IVF (hpi) was similar between preovulatory follicular maturity treatments (*P* ≥ 0.24, Table 1). Maturation of COCs with exposure to follicular fluid from preovulatory follicles of greater or lesser maturity for 24 h IVM did not affect the ability of the oocytes to cleave or develop into a blastocyst (*P* ≥ 0.28, Table 1). Embryo quality was not influenced by preovulatory follicle maturity treatment (*P* = 0.25). No differences in percentage of embryos reaching the 2-cell, 4-cell, or 8–16 cell stage were observed when cleavage was assessed at 73 hpi (*P* ≥ 0.12). At some point between 73 and 193 hpi, embryos from the greater follicle maturity treatment experienced a more moderate rate of development because mean development stage at 193 hpi was lower in embryos originating from oocytes exposed to follicular fluid from preovulatory follicles of greater vs lesser maturity (*P* = 0.01, Table 1).

**Table 1.** Embryo development following COC treatment with follicular fluid from preovulatory follicles of greater or lesser physiological maturity for 24 h IVM.

				Cleavage				Development		
		~16^a^hpi		~73 hpi				~193 hpi		
^b^IVM treatments	^c^Total COC	^d^PZ Rcd (%)	Lysed (%)	Cleaved (%)	2-cell (%)	4-cell (%)	8- to 16-cell (%)	Blastocyst (%)	Quality	Stage
Greater follicle maturity	1,815	93.8	2.6	73.9	10.1	13.6	76.3	26.2	1.4	6.5
Lesser follicle maturity	1,815	92.7	1.7	73.5	7.4	18.8	73.9	24.8	1.3	6.7
	SEM	±1.5	±0.53	±2.8	±1.7	±2.5	±2.8	±2.8	±0.08	±0.09
	^e^ *P*-value	0.50	0.24	0.91	0.21	0.12	0.56	0.28	0.25	**0.01**
^f^FBS reference	1,275	95.8	3.2	77.8	10.6	18.7	71.0	22.9	1.4	6.5

^a^hpi, hours post in vitro fertilization.

^b^IVM, in vitro maturation; treatments were applied during 24 h IVM and consist of oocyte maturation media (OMM) supplemented with 20% follicular fluid from preovulatory follicles of greater or lesser physiological maturity, based on serum estradiol concentration, at the time of gonadotropin releasing hormone administration to induce the luteinizing hormone surge. Serum estradiol concentration was >2 fold higher in all animals of greater vs lesser follicle maturity (11.8 ± 0.37 pg/mL vs 3.7 ± 0.32 pg/mL, respectively).

^c^COC, cumulus-oocyte complex.

^d^PZ Rcd, putative zygotes recovered.

^e^ *P*-value, *P*-value for statistical analyses using greater and lesser physiological maturity treatments only.

^f^Lab reference for embryo development; 20% fetal bovine serum supplemented OMM.

Values represented as least square means.

### Metabolome profiles of OMM supplemented with follicular fluid of preovulatory follicles with greater vs lesser physiological maturity

A total of 112 and 128 metabolites, respectively, were detected in preovulatory follicular fluid supplemented OMM samples collected before and after IVM (Table S1 and S2). Of these metabolites, 111 were detected in both media timepoints, while 2 and 18 metabolites were detected in only before or after maturation samples, respectively.

**Fig. 1.**
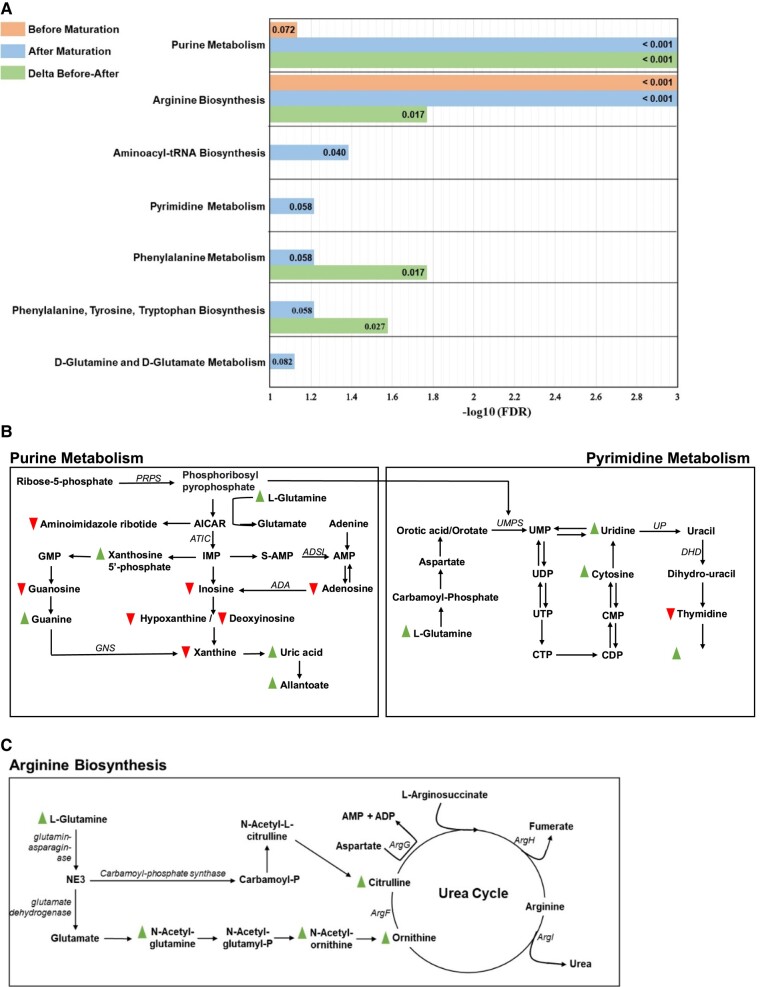
A) Pathways enriched with metabolites differentially abundant in oocyte maturation media supplemented with preovulatory follicular fluid of greater vs lesser physiological maturity. Before maturation is represented by the top bar in each pathway, after maturation by the middle bar, and change in metabolite abundance from before to after maturation (delta) by the bottom bar in each pathway. Actual FDR is written in each bar. B) Schematic of purine and pyrimidine metabolism. C) Schematic of arginine biosynthesis. In (B and C), triangles represent metabolites that were more abundant in the greater follicle maturity treatment. Inverted triangles represent metabolites that were less abundant in the greater follicle maturity treatment.

Sparse partial least squares discriminate analyses of metabolome profiles clustered OMM samples based on preovulatory follicle maturity treatment (Fig. S1). Forty-eight metabolites (48/112 detected, 43%) differed in abundance between greater and lesser follicle maturity treatments at onset of oocyte maturation. Forty (83%) of differentially abundant metabolites had increased, and 8 (17%) had decreased abundance in the greater follicle maturity treatment (Table S1). Pathway analysis demonstrated enrichment of the pathways “Arginine biosynthesis” and “Purine metabolism” with metabolites influenced by preovulatory follicle maturity classification at the onset of oocyte maturation (FDR < 0.08, Fig. 1).

In OMM samples collected at the conclusion of 24 h IVM, 66 metabolites (66/128 detected, 52%) differed in abundance between greater and lesser preovulatory follicle maturity treatments. Fifty-four (82%) of differentially abundant metabolites were increased, and 12 (18%) were decreased in abundance in the greater follicle maturity treatment (Table S2). Pathway analysis of differentially abundant metabolites in OMM after conclusion of oocyte maturation demonstrated enrichment of the pathways “Purine metabolism”, “Arginine biosynthesis”, “Aminoacyl-tRNA biosynthesis”, “Pyrimidine metabolism”, “Phenylalanine metabolism”, “Phenylalanine, tyrosine, and tryptophan metabolism”, and “D-Glutamine and D-glutamate metabolism” with media metabolites influenced by preovulatory follicle maturity (FDR < 0.09, Fig. 1).

Of the 66 metabolites that differed between preovulatory follicle maturity treatments in OMM collected after 24 h IVM, 30 (45%) were uniquely affected by treatment in post maturation OMM. These metabolites did not differ between treatments at the onset of oocyte maturation and represent a change in COC metabolism due to preovulatory follicle maturity. Twenty-seven (90%) of differentially abundant metabolites unique to OMM after 24 h IVM were increased and 3 (10%) were decreased in abundance in the greater follicle maturity treatment. Pathway analysis of differentially abundant metabolites uniquely identified in postmaturation media demonstrated enrichment of the pathways “Purine metabolism”, “Phenylalanine, tyrosine, and tryptophan biosynthesis”, and “Aminoacyl-tRNA biosynthesis” (FDR < 0.10).

Also related to changes in COC metabolism due to treatment, 30 metabolites (28% of those identified in OMM both before and after 24 h IVM) displayed differential change in abundance from onset to completion of oocyte maturation based on preovulatory follicle maturity treatment (Table S3). Pathway analysis demonstrated enrichment of the pathways “Purine metabolism”, “Aminoacyl-tRNA biosynthesis”, “Phenylalanine metabolism”, and “Phenylalanine, tyrosine, and tryptophan metabolism” with metabolites with differential change in abundance based on preovulatory follicle maturity classification (FDR < 0.03, Fig. 1).

### Impact of preovulatory follicle maturity treatment during oocyte maturation on abundance of metabolites in media conditioned by developing embryos

**Fig. 2.**
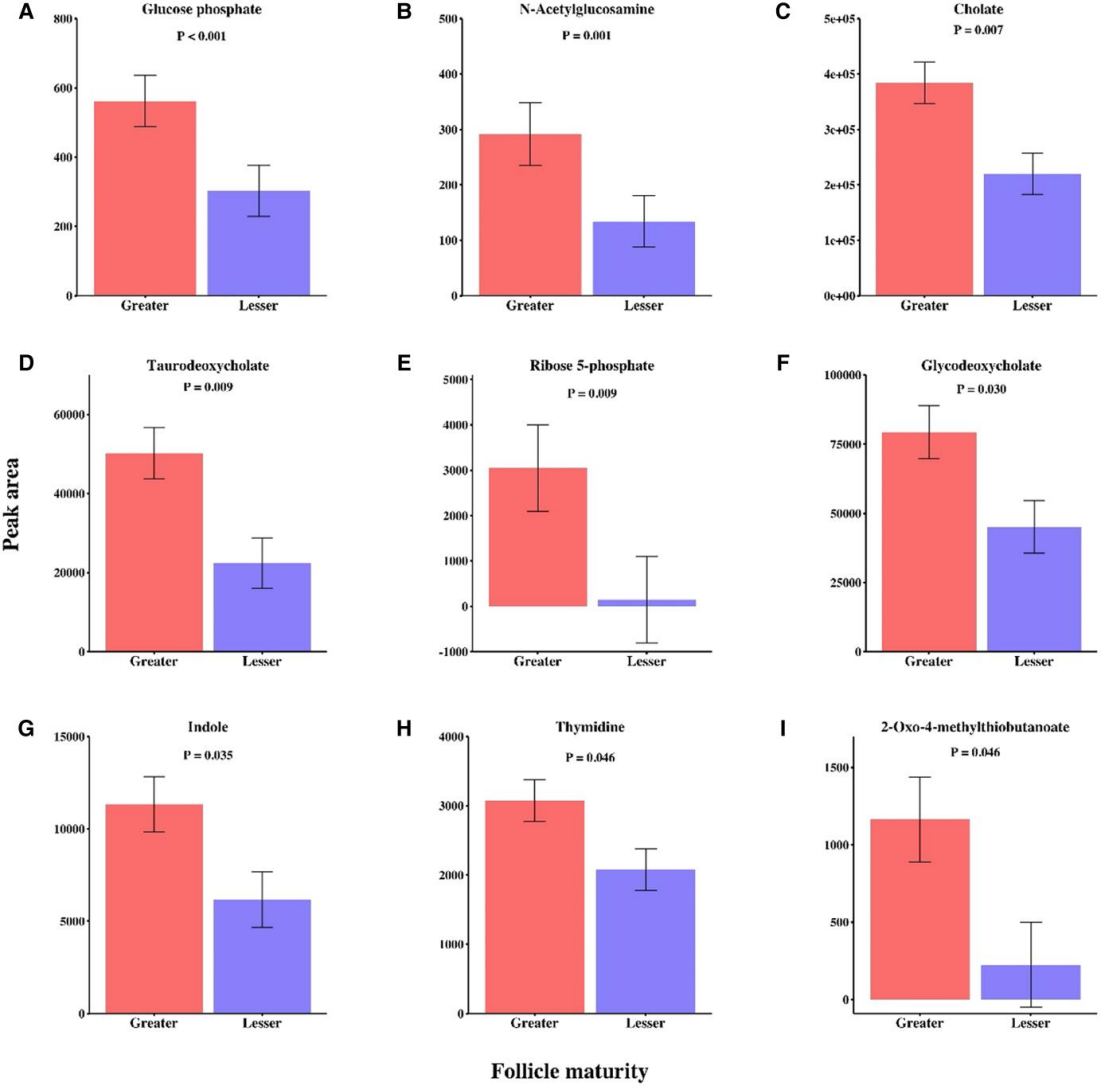
A–I) Bar chats representing the nine differentially abundant metabolites in conditioned media following embryo development of oocytes supplemented with follicular fluid from preovulatory follicles of greater vs lesser maturity during 24 h in vitro maturation.

Sixty-one metabolites were detected in embryo conditioned media collected on day 8 postfertilization. Nine metabolites (15%) differed in abundance between greater and lesser follicle maturity treatments (Fig. 2). All differentially abundance metabolites had greater abundance in media from blastocysts originating from oocytes matured with 20% follicular fluid from preovulatory follicles of greater physiological maturity.

## Discussion

Results of this study demonstrated profound effects of preovulatory follicle maturity on metabolism of maturing bovine COCs, despite minimal differences in embryo development. In vitro supplementation of maturing COCs with 20% follicular fluid from preovulatory follicles of greater vs lesser maturity before a GnRH-induced LH surge led to extensive differences in metabolite usage or production during oocyte maturation. This was followed by a direct effect on embryo development stage at 8 days postinsemination and differential abundance of nine metabolites in media conditioned by developing embryos. Consequences of follicle maturity treatment on COC metabolism and subsequent embryo stage are well aligned with previous studies that demonstrated reduced embryo stage 7 days post fixed-time artificial insemination (12, 13) and increased metabolic preparation of the oocyte 18–20 h post GnRH administration (17, 18) in preovulatory follicles of greater physiological maturity. This said, results described within are the first to move beyond observed relationships and demonstrate a direct effect of follicle maturity on the maturing oocyte.

Findings of the current study suggest that oocytes treated during 24 h IVM with preovulatory follicular fluid of greater vs lesser physiological maturity were metabolically programmed for a moderate rate of embryo development. While there were no differences in embryo stage at 73 hpi, rate of development moderated in embryos derived from oocytes of the greater vs lesser follicle maturity treatment at some point between 73 h and 8 days postinsemination. In support of the quiet embryo hypothesis and goldilocks theory, moderate rate of embryo development has previously been associated with improved fertility (20–25). There is little doubt that embryos developing at an abnormally slow rate are less fertile (23, 24, 26, 27); however, there is growing knowledge that an optimal vs hastened rate of embryo development results in highest fertility. In humans, embryos of intermediate development speed had increased blastocyst formation, implantation rate, and pregnancy rate compared to embryos with slow or rapid development (28–30). Results described herein suggest that rate of embryo development may be influenced by the readiness of the intrafollicular environment to provide optimal metabolic support of the maturing oocyte, and that bioavailability of metabolites during oocyte maturation metabolically programs the oocyte for optimal embryo development rate. This said, interpretation of moderate embryo development rate on competence of the embryo for pregnancy should be cautiously interpreted since embryos were not transferred in the current study.

Profound differences in the metabolome of conditioned OMM and embryo development media were observed when maturing oocytes were supplemented with 20% follicular fluid from preovulatory follicles of greater vs lesser physiological maturity prior to the GnRH-induced LH surge. Although initial differences in OMM metabolome between greater and lesser preovulatory follicle maturity treatments were observed, treatment of OMM with 20% preovulatory follicular fluid of greater maturity led to robust changes in 42 metabolites in OMM collected after 24 h IVM that either were not different at maturation onset or had differing degrees of accumulation or decrease from onset to completion of IVM. Interestingly, a majority of metabolites affected by preovulatory follicle maturity treatment were higher in abundance after IVM with follicular fluid from greater maturity follicles. Such results follow previous trends noted by our team, in which metabolites in follicular fluid collected 18 h post GnRH-induced LH surge had greater abundance with increasing preovulatory follicle maturity (18, 19).

Based on previous accounts of increased metabolites critical for glucose metabolism in follicular fluid from preovulatory follicles of greater maturity (18, 19), it was not surprising that treatment linked differences in abundance of individual metabolites related to glucose metabolism were observed in OMM. Pyruvate, alpha-ketoglutarate, 2-dehydro-d-glutamate, citrate/isocitrate, and glucose phosphate were increased in the greater follicle maturity treatment before the onset of oocyte maturation. All but pyruvate maintained this treatment effect in OMM samples collected after 24 h IVM. Furthermore, a greater degree of increase in 2-dehydro-D-gluconate, alpha-ketoglutarate, D-gluconate, and lactate was observed between OMM collected before and after IVM in the greater follicle maturity treatment. Results suggest improved capacity for glucose metabolism in COCs submitted to maturation with 20% follicular fluid of greater physiological maturity. Increased lactate accumulation in the greater follicle maturity treatment during IVM is especially interesting, as previous in vitro studies suggest increased cumulus cell glycolytic activity, observed by increased lactate production, in COCs of greater developmental competence (31). Additionally, glycolytic enzymes and lactate dehydrogenase were upregulated in cumulus cells collected at 20 h post-GnRH administration in preovulatory follicles of greater vs lesser physiological maturity (17). The oocyte relies on energetic support from companion cumulus cells and the surrounding follicular environment since it does not have capacity for glycolytic conversion of glucose into pyruvate to fuel oxidative phosphorylation (32). This said, mitochondria within maturing oocytes have a unique phenotype, with fewer cristae, that reduces their capacity for oxidative phosphorylation (33, 34). Therefore, alternative routes for intraoocyte energy production, such as the pentose phosphate pathway, adenosine salvage pathway, and fatty acid breakdown, have been proposed.

The pentose phosphate pathway is an alternative route of glucose metabolism that generates metabolic intermediates such as nicotinamide dinucleotide phosphate (NADPH) to serve as precursors for nucleotide and amino acid biosynthesis. Metabolic fate of glucose was not determined in the current study; however, impacts of follicle maturity treatment on abundance of purines, pyrimidines, and amino acids (AA) may indicate differential regulation of glucose metabolism through glycolysis or pentose phosphate due to differing follicular fluid milieu associated with preovulatory follicle maturity. Purine and pyrimidine nucleotides are essential cellular components involved in energy metabolism, oxidative balance, and nucleic acid replication, transcription, and translation (35, 36). Regardless of follicular fluid origins, levels of all differentially abundant purine metabolites increased in OMM from the onset to completion of 24 h IVM. Under normal cellular conditions, enzymes that control purine metabolism are regulated in a manner that maintains a balanced ratio between purine synthesis and breakdown. Breakdown of purines leads to the production of uric acid, which was greater in abundance in conditioned OMM supplemented with 20% follicular fluid of greater preovulatory follicle maturity. Increased OMM accumulation of uric acid during 24 h IVM may indicate improved mechanisms for ensuring adequate balance of reactive oxygen species (ROS) in the greater follicle maturity treatment. Uric acid is a potent antioxidant in the extracellular environment that also acts as a pro-oxidant in the cell by stimulating NADPH oxidase (37). Precise regulation of ROS accumulation within the preovulatory follicle and environment of the maturing oocyte is essential for ovulation, oocyte maturation, and high fertility. Although excessive accumulation of ROS without sufficient regulation is detrimental to oocyte maturation (38, 39), it is thought that a maintained level of ROS could be beneficial for critical developmental timepoints such as induction of meiotic resumption (39). As reviewed by Jamil and others in 2020, ideal concentration of ROS is highly dependent on developmental stage of the oocyte or embryo. Finely tuned increases in ROS during oocyte maturation may be a byproduct of superior intrafollicular environment of preovulatory follicles of greater physiological maturity (40).

Increased abundance of uric acid and a primary decrease of intermediate metabolites of uric acid production in the greater follicle maturity treatment may indicate differences in nucleotide recycling between COCs submitted to 24 h IVM in OMM supplemented with either greater or lesser maturity follicular fluid. In alternative to their production of uric acid, purine bases can be salvaged and converted back to their triphosphate form to conserve energy (41). If metabolic capacity is limited in lesser mature follicles, nucleotide salvage vs production of uric acid may be necessary to ensure adequate energy is available to meet the maturing oocyte's needs. Impact of follicular fluid treatment on adenine and guanine nucleotide breakdown during in vitro oocyte maturation is especially intriguing due to the role of their cyclic versions, cyclic adenosine monophosphate and guanosine 3′,5′-cyclic monophosphate, in regulation of oocyte meiotic arrest and resumption (42–44). Differential accumulation of adenine and guanine nucleotide by-products may be related to kinetics or success of oocyte maturation; however, experimental design of the current study limits any interpretations related to an effect of treatment on oocyte maturation to observed similarities in embryo cleavage or blastocyst rate.

In addition to glutamine, which is a precursor for purine synthesis, the AA asparagine, citrulline, histidine, ornithine, phenylalanine, tryptophan, and tyrosine had increased abundance in conditioned OMM from the greater follicle maturity treatment. Abundance of n-acetyl-beta-alanine, n-acetylglutamine, n-acetylglutamate, and n-acetylornithine also followed this trend. Uptake of glutamine and other AA is essential for oocyte metabolic preparation for embryo development that is dependent on somatic follicular components (45). Turnover of AA has been proposed as a marker for developmental competence of oocytes and embryos (46, 47). Increased abundance of numerous AA in post oocyte maturation OMM supplemented with greater maturity follicular fluid suggests more efficient amino acid metabolism in follicles of greater physiological maturity. Increased glutamine in the greater maturity treatment could greatly improve oocyte competence for embryo development, as glutamine is a cellular substrate for nucleotide and protein synthesis, glutathione, NADPH, and many other biological pathways essential to maintain cellular integrity, oxidative balance, and cellular adaption to physiological stressors (48–50). Alternatively, glutamine can be utilized through its entry into the tricarboxylic acid cycle through conversion to glutamate and then alpha-ketoglutarate for energy production (51). Arginine is also a necessary amino acid during the maturation period of the bovine COC (52), and several AA in the arginine biosynthesis pathway that were influenced by follicle maturity play important roles in protein production to support overall health and function of the cell (53). Arginine is also a precursor of nitric oxide (54). Although high levels of nitric oxide are damaging, inhibition of nitric oxide signaling results in apoptosis of embryonic cells (55).

Interestingly, nine metabolites differed in abundance within media conditioned during embryo culture of fertilized oocytes treated with follicular fluid from preovulatory follicles of greater and lesser maturity. Seven of the nine metabolites that were affected by follicular fluid treatment in conditioned embryo culture media were also impacted by treatment during oocyte maturation. Glucose phosphate, cholate, glycodeoxycholate, taurodeoxycholate, and thymidine were affected by follicle maturity treatment in conditioned embryo development media as well as OMM collected before and after 24 h IVM. Further, indole and n-acetylglucosamine were differentially abundant in greater or lesser maturity follicular fluid treatments in postmaturation OMM and conditioned embryo development media. Embryo culture media was made as a single batch for each replicate of in vitro embryo production; thus, the media for development of each treatment was identical when plated. Since COCs were removed from treated OMM for fertilization and putative zygotes were washed before placement into embryo culture media, metabolic programming of developing embryos due to preovulatory follicle maturity treatment during oocyte maturation was the most likely cause of any differences in the metabolome of conditioned embryo development media. Ability to make inferences on the impact of differential embryo production or usage of these metabolites is limited, but increased abundance of ribose phosphate is especially relevant to results observed in OMM as ribose phosphate is a byproduct of the pentose phosphate pathway that is utilized for purine and amino acid synthesis (56). Increased levels of ribose phosphate may indicate a longstanding effect of greater maturity follicular fluid on pentose phosphate pathway activity, purine metabolism, and amino acid synthesis in the embryo that promotes optimal rate of metabolism and development.

Increased abundance of numerous metabolites in OMM supplemented with greater follicle maturity follicular fluid, followed by increased metabolite abundance or greater degree of metabolite increase in OMM collected after 24 h IVM suggests improved metabolic support of the maturing COC. We speculate that greater follicle maturity follicular fluid better prepares the oocyte for maturation and subsequent embryo development. As such developing embryos undergo cell divisions at a more efficient rate and thereby utilize fewer metabolic resources in embryo development media. Although no apparent difference in blastocyst rate or quality were observed, the increased speed of embryo development in blastocysts treated with follicular fluid from preovulatory follicles of lesser maturity may be due to compensatory increases in metabolism following oocyte maturation in a subpar metabolic milieu. Lack of observed differences in embryo cleavage or blastocyst development and quality, compared to previously published in vivo results is not surprising. Follicle maturity treatments in the current study only occurred during oocyte maturation, and differences between treatments were limited to 20% follicular fluid supplementation of otherwise identical medium. Within the animal, the COC's maturation environment is 100% preovulatory follicular fluid, and compounding impacts of follicle maturity on the oocyte itself and the uterine environment would undoubtedly exemplify any effects of follicle status observed in vitro. This said, such confounding factors make it difficult to infer causality between preovulatory follicle physiological status and the follicle's ability to support oocyte preparation for embryo development.

Procedures of the current study remove confounding in vivo derived factors and allow for direct causality to be tested between the preovulatory follicular fluid milieu and oocyte metabolism and competence for early embryo development. As such, the results described within are the first to demonstrate causality between bovine follicular fluid environment associated with preovulatory follicle maturity and both metabolic and developmental competence of the oocyte. Results highlight the ever-important maternal legacy of intrafollicular support of oocyte preparation for embryo development and demonstrate substrates within OMM that hold promise to improve developmental competency of the maturing oocyte.

## Materials and methods

### Synchronization and collection of preovulatory follicular fluid samples

**Fig. 3.**
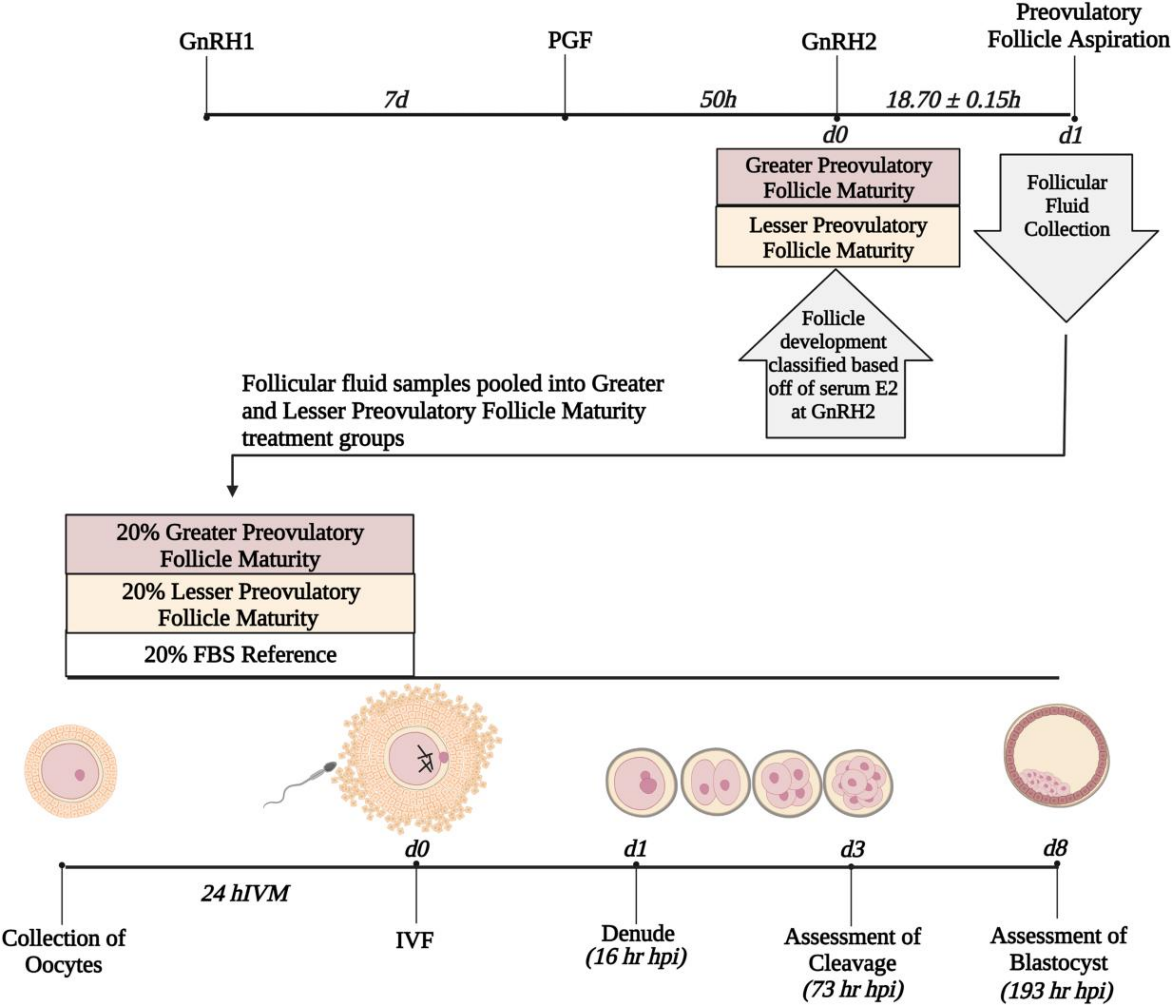
Schematic of preovulatory follicular fluid collection, treatment classification, and in vitro maturation treatment scheme. Preovulatory follicular fluid samples were collected 18.70 ± 0.15 h after gonadotropin releasing hormone administration to induce the preovulatory luteinizing hormone surge (GnRH2). Follicles were classified as greater or lesser physiological maturity based on serum estradiol (E2) at GnRH2. Immature bovine cumulus-oocyte complexes were randomly placed into treatment groups to mature at 38.5°C, 5.5% CO_2_, 21.0% O_2_ for 24 h. Treatment groups consisted of oocyte maturation media (OMM) supplemented with 20% follicular fluid (FF) of greater physiological maturity (Greater), 20% FF of lesser physiological maturity (Lesser), and 20% fetal bovine serum (FBS, reference for lab). After 24 h in vitro maturation, in vitro fertilization (IVF) was performed. At 16 h post IVF (hpi), putative zygotes were denuded of associated cumulus and spermatozoa. At 73 hpi, cleavage of putative zygotes was recorded. At 193 hpi, blastocyst development was recorded. For each treatment, 60 µL OMM was collected before and after 24 h IVM and after blastocyst assessment for metabolome analysis. GnRH1, gonadotropin-releasing hormone first administration; PGF, prostaglandin F2α.

All procedures involving animals were approved by the University of Tennessee Institutional Animal Care and Use Committee. Development of a preovulatory follicle was synchronized in postpartum, lactating Angus cows (*n* = 136), subjected to a 7-day CO-Synch according to procedures detailed in (18) and outlined in Fig. 3. All follicles >7 mm in diameter were recorded utilizing a Samsung HM70A ultrasound with corresponding CF4–9 convex probe at initial GnRH administration (GnRH1), prostaglandin F2α (PGF) administration, second GnRH administration to induce the LH surge (GnRH2), and follicle aspiration to collect preovulatory follicular fluid. Follicle size was calculated by averaging the largest lengthwise internal diameter and the largest transverse internal diameter, formulating a cross-hatch pattern. Blood samples were taken at GnRH1, PGF, GnRH2, and follicle aspiration via the coccygeal tail vein/artery. Blood samples were allowed to clot at room temperature for 1 h before being refrigerated at 4°C for 24 h. Samples were then centrifuged at 1,200×g for 25 m at 4°C. Serum supernatant was stored in borosilicate glass tubes (VWR, Radnor, PA, USA) and stored at −20°C.

Estrotect™ patches were utilized to identify and remove animals that displayed estrus during synchronization to increase confidence that preovulatory follicular fluid samples originated from cows that required GnRH2 administration to induce the preovulatory gonadotropin surge. Patches were scored on a scale of 0 to 4 with 0 indicating a missing patch (patch = 0), 1 indicating <25% rubbed off (no estrus), 2 indicating 25 to 50% rubbed off (no estrus), 3 indicating 50 to 75% rubbed off (estrus), and 4 indicating >75% rubbed off (estrus).

Preovulatory follicular fluid was collected from each cow via transvaginal follicle aspiration 18.70 ± 0.15 h after GnRH2 administration to induce the LH surge. The follicular fluid collected (*n* = 103) was moved to a 1.7 mL tube (VWR, Radnor, PA, USA) and centrifuged at 500×g at 4°C for 5 m to pellet any cellular debris. Follicular fluid supernatant was removed from the pellet carefully and was stored in borosilicate glass tubes for hormone assays and 2 mL cryovials for potential use in the in vitro supplementation study. Follicular fluid samples placed in borosilicate glass tubes were stored at −20°C and samples stored in 2 mL cryovials were snap frozen with liquid nitrogen and stored at −80°C.

### Serum and follicular fluid radioimmunoassay

Serum samples collected at GnRH1, PGF, GnRH2, and follicle aspiration were submitted to estradiol radioimmunoassay utilizing an approach validated by Kirby et al. (57). Serum estradiol intra-assay and inter-assay coefficients of variation (CV) averaged 3.45 and 8.74%, respectively. Assay sensitivity was 1.03 pg/mL. Follicular fluid samples were diluted from 1:20 to 1:2500 for estradiol assay and 1:5 to 1:20 for progesterone assay. The DetectX® Serum 17β-Estradiol ELISA Kit (sensitivity = 2.21 pg/mL, Arbor Assays, Ann Arbor, MI, USA) was used to quantify follicular fluid free estradiol concentration. Follicular fluid intra-assay and inter-assay CV for free estradiol averaged 4.5 and 8.11%, respectively. Serum (collected at GnRH1, PGF, GnRH2, follicle aspiration) and follicular fluid progesterone was assessed using the ImmuChem Progesterone Double Antibody Radioimmunoassay Kit (sensitivity = 0.11 ng/mL, MP Biomedicals, Costa Mesa, CA, USA), as previously validated by Pohler et al. (58). Serum progesterone intra-assay and inter-assay CV averaged 5.42 and 5.72% respectively. Follicular fluid progesterone intra-assay and inter-assay CV averaged 4.23 and 5.03%, respectively.

### Preovulatory follicle maturity classification

Serum estradiol concentration at the administration of GnRH2 to induce the preovulatory LH surge was used to categorize follicular fluid samples into upper, middle, and lower thirds for preovulatory follicle maturity. Follicular fluid samples were excluded from the study if they had less than 100 µL of total follicular fluid available for the supplementation study, any noted contamination (e.g. digesta, blood), or a follicular fluid estradiol: progesterone ratio >3.12 (*n* = 43). Samples remaining from cows in the upper third of serum estradiol values at GnRH2 (9.5–15.4 pg/mL) were classified as originating from follicles with greater physiological maturity and used for the greater follicle maturity treatment (*n* = 17). Samples remaining from cows in the lower third of serum estradiol at GnRH2 (1.9–4.9 pg/mL) were classified as originating from follicles with lesser physiological maturity and used for the lesser follicle maturity treatment (*n* = 12). Cow variables and hormone profiles were carefully assessed to ensure that follicular fluid from preovulatory follicles of greater and lesser maturity originated from animals that were otherwise as similar as possible (Table 2). Follicular fluid samples from each follicle maturity category were then pooled to form a homogenous aliquot for each treatment group. After pooling, follicular fluid was aliquoted for single thaw use during the IVM study.

**Table 2.** Phenotypic parameters of cows with a preovulatory follicle of greater or lesser physiological maturity at GnRH administration.

	^a^Follicle development		
Parameter	Greater *n* = 17	Lesser *n* = 12	*P*-value
Serum E2 at GnRH2 (pg/mL)	11.8 ± 0.37	3.7 ± 0.32	<0.01
Serum P4 at GnRH2 (ng/mL)	0.3 ± 0.09	0.17 ± 0.11	0.36
Preovulatory follicle diameter at GnRH2 (mm)	13.5 ± 0.31	11.8 ± 0.45	0.02
Serum E2 at FA (pg/mL)	4.9 ± 0.48	3.1 ± 0.21	0.02
Serum P4 at FA (ng/mL)	0.2 ± 0.05	0.3 ± 0.06	0.76
Preovulatory follicle diameter at FA (mm)	13.4 ± 0.31	11.6 ± 0.45	0.03
Follicular fluid E2 at FA	66.5 ± 6.23	43.9 ± 7.42	0.03
Follicular fluid P4 at FA	97.8 ± 10.00	78.2 ± 11.90	0.22
Follicular fluid E2:P4 ratio at FA	0.8 ± 0.13	0.8 ± 0.15	0.95
Cow age (years)	5.4 ± 0.59	5.5 ± 0.70	0.87
Cow BCS	5.7 ± 0.25	6.3 ± 0.30	0.14
Cow days postpartum	57.2 ± 0.88	58.1 ± 1.05	0.51

E2, estradiol; GnRH2, gonadotropin releasing hormone administration to induce the preovulatory gonadotropin surge; P4, progesterone; FA, follicle aspiration; BCS, body condition score.

^a^Data are presented as mean + standard error of the mean.

### In vitro production of embryos

Chemicals and reagents were purchased from MilliporeSigma (Merck Group, St. Louis, MO, USA) unless stated otherwise. The same batches of follicle stimulating hormone (FSH, Folltropin®, Vetrepharm Canada INC, London, ON, Canada) and fetal bovine serum (FBS, BioWhittaker, Walkerxville, MD, USA) were used for the entirety of the study. Medium-199 without phenol red, penicillin-streptomycin, and gentamycin were purchased from Invitrogen (ThermoFisher Scientific Corporation, Carlsbad, CA, USA). Tyrode's Lactate and Modified Tyrode's Albumin Lactate Pyruvate (TALP) base-media (HEPES-TALP, IVF-TALP, and Sperm TALP) were prepared according to Parrish et al (59), and Potassium Simplex Optimized Medium was prepared (58), but modified to contain 0.5% BSA, 10 mM glycine, 1 mM L-glutamine, 1×nonessential AA, 50 U/mL penicillin, and 50 µg/mL streptomycin (mKSOM) (60).

General methods for in vitro production of embryos (Fig. 3) were performed as previously described in (61) using abattoir derived ovaries. Cumulus-oocyte complexes with at least three layers of cumulus cells and homogenous ooplasm underwent IVM at 38.5°C in 5.5% CO_2_, 21% O_2_ for 24 h. For each replicate, approximately 30 COCs were matured per 0.5 mL OMM in a 4-well culture dish (Nunc™, ThermoFisher Scientific, Waltham, MA, USA). Temperatures maintained within the incubator system were verified before and during the experiment using mercury thermometers sealed in media-filled bottles. After 24 h IVM, COCs underwent IVF using frozen-thawed Percoll-prepared semen (500,000 motile sperm/mL). Each experimental replicate and treatment used the same bull for IVF. Putative zygotes were denuded of associated cumulus cells and spermatozoa 14–17 hpi and placed into 500 µL of KSOM to culture at 38°C in 5.5% CO_2_, 7.0% O_2_. Cleavage and blastocyst development were assessed ~ 73 and 193 hpi. Stage and quality scores were also assigned at 193 hpi as described by Rispoli et al. (62).

### Study design

The in vitro study was replicated across 11 different COC collection days (Fig. 3). Plates for IVM were prepared 24 h before COC collection. Each treatment plate was then supplemented with follicular fluid from preovulatory follicles of greater or lesser maturity. The greater follicle maturity treatment plate contained OMM supplemented with 20% follicular fluid assigned as greater follicle maturity and the lesser follicle maturity treatment plate contained OMM supplemented with 20% follicular fluid assigned as lesser follicle maturity. For each replicate, a development reference plate was also prepared that contained OMM supplemented with 20% FBS. Before incubation to equilibrate the media, 60 µL of media from each treatment was snap frozen and stored at −80°C for metabolomic analysis (before maturation OMM).

Cumulus-oocyte complexes were randomly allocated to greater follicle maturity treatment, lesser follicle maturity treatment, or FBS reference plates for 24 h IVM. At the time of fertilization, when OMM was aspirated off COCs to be replaced with IVF-TALP, spent supplemented OMM from each treatment was placed into a 2 mL Eppendorf and centrifuged at 1500×g for 3 min at 4°C to pellet any cellular debris unintentionally collected during aspiration. Then, 60 µL of spent OMM from each treatment was snap frozen and stored at −80°C for metabolomic analysis (after maturation OMM). After denudement of cumulus and spermatozoa, putative zygotes were submitted to identical culture methods detailed above. In replicates 7–11, conditioned embryo development media was collected at 193 hpi and processed identically to after maturation OMM.

### UHPLC-HRMS metabolomics

Sixty µL samples of before maturation OMM, after maturation OMM, and conditioned embryo development media were submitted for metabolomic analysis according to procedures outlined previously by our team (7, 8, 18, 19, 63). Metabolites from each sample were extracted with 0.1 M formic acid using a solution of 20:40:40 water/methanol/acetonitrile. Metabolites were separated on a Synergi Hydro RP, 2.6 µm, 100 mm × 2.1 mm column (Phenomenex, Torrance, CA, USA) using a tributylamine ion pairing reagent and a mobile phase. The solvents for the mobile phase to propel metabolites were as follows: (i) 97:3 water:methanol with 11 mM tributylamine and 15 mM acetic acid, (b) 100% methanol. Solvent gradients from 0 to 5 min was (i) 100%, (ii) 0%. Solvent gradients from 5 to 13 min was (i) 80% and (ii) 20%. Solvent gradients from 13 min to 15.5 min was (i) 45% and (ii) 55%. Solvent gradients from 15.5 to 19 min was (i) 5% and (ii) 95%. Lastly, solvent gradients from 19 to 25 min were (i) 100% and (ii) 0% with an overall flow rate of 0.2 mL/min. The separated metabolites were ionized in the negative mode polarity with an electrospray ionization probe and an Exactive™ Plus Orbitrap™ mass spectrometer (Thermo Fischer Scientific, Waltham, MA, USA) with a resolution of 140,000 was used for full scan mass analysis (64).

Raw data from UPLC-HRMS were converted to the open source mzML format (msconvert software, ProteoWizard package) and were then processed within Metabolomic Analysis and Visualization Engine (MAVEN, mzroll software, Princeton University). MAVEN software was used for nonlinear retention time correction, peak picking alignment across samples, and peak intensities integration. Known metabolites in the samples were identified by matching their chromatographic retention time and exact masses within ± 5 ppm mass tolerance to an in-house standard library of metabolites with known exact masses and retention time. Peak shape, signal-to-noise ratio, and retention time were all used in conjunction to select metabolites. Preprocessed peak data tables processed in MAVEN were used for statistical analysis.

### Statistical analyses

Statistical procedures were performed using R Studio (version 4.1.2, RStudio Team 2021, Boston, MA, USA) and SAS 9.4 (SAS Institute Inc., Cary, NC, USA). All data were tested for normality using Shapiro–Wilk. All statistical analyses were considered significant if *P* < 0.05, and all data are reported as least square means ± standard error of the mean (SEM). Initial confirmations of cow parameters and hormone profiles between preovulatory follicle maturity classifications were assessed using analysis of variance in R Studio. Embryo development data were analyzed using a randomized block design, blocking on day of COC collection, using a linear mixed model in SAS (PROC MIXED). The experimental unit for the in vitro study was the 4-well Nunc plate where grouped COCs underwent maturation, as follicle maturity treatments were applied to a plate rather than individual oocytes. Differences between greater and lesser follicle maturity treatments were determined using protected Fisher's Least Square Differences. The analyses for metabolomics were performed in R Studio (nlme). Change in metabolite abundance during maturation was calculated by subtracting before maturation OMM values from after maturation OMM values. A mixed effects linear model, blocking on day of COC collection, was utilized for all media metabolome analyses. For metabolite analyses of conditioned embryo development media, covariates of average stage, quality, putative zygotes submitted to KSOM, and number of blastocysts per treatment plate were also included. Type III Sum of Squares was then utilized to determine the significance of preovulatory follicle maturity treatment on metabolite abundances in each media type. Metabolites that were significantly affected by treatment in before maturation OMM, after maturation OMM, and change in OMM were input into MetaboAnalyst 5.0 to perform Kyoto Encyclopedia of Genes and Genomes pathway enrichment analyses. Pathway significance was established at Benjamini–Hochberg FDR < 0.10.

## Supplementary Material

pgae181_Supplementary_Data

## Data Availability

The data produced in this research is publicly available in the Dryad repository (doi:10.5061/dryad.d2547d88x).
