# Supplementary material for: Impact of preovulatory follicle maturity on oocyte metabolism and embryo development
Source: PNAS Nexus. 2024 Apr 30;3(5):pgae181. doi: 10.1093/pnasnexus/pgae181 (PMC11095542; doi:10.1093/pnasnexus/pgae181)
Supplement: pgae181_Supplementary_Data [file pgae181_supplementary_data.pdf]

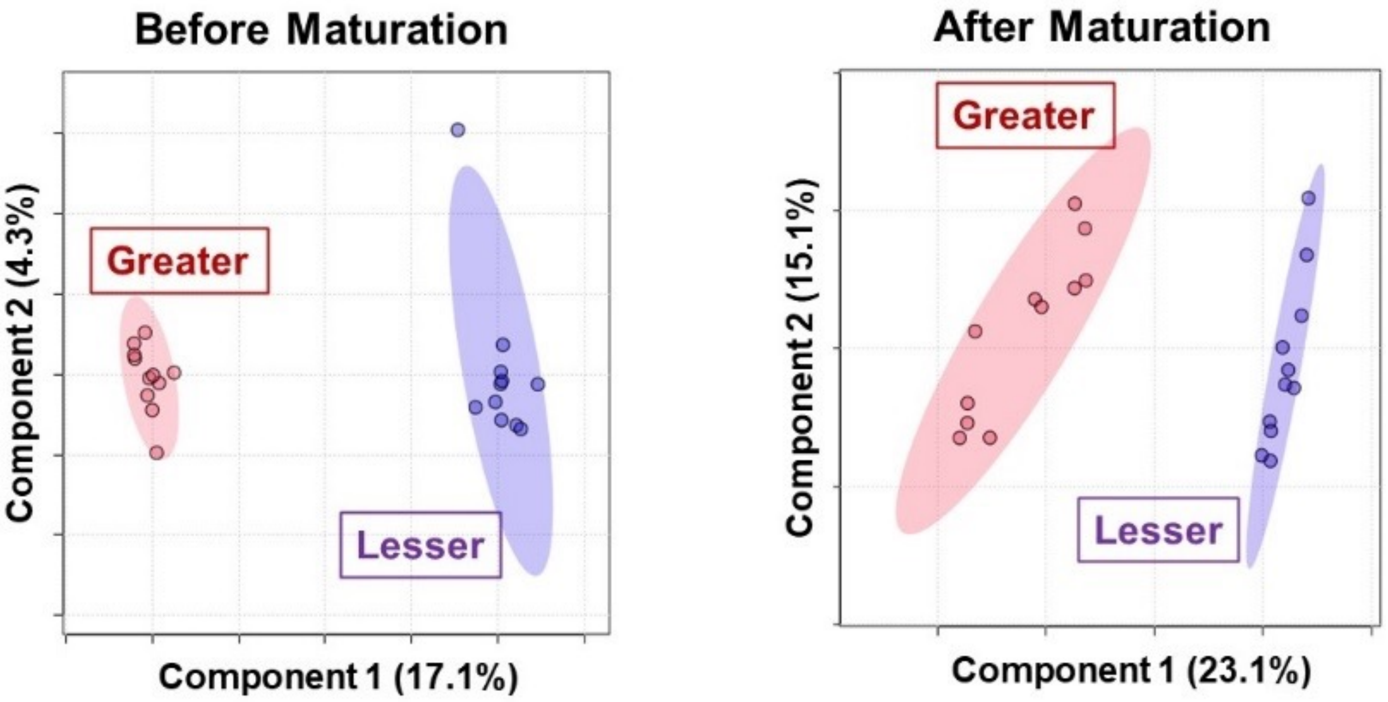

Figure S1. Sparse partial least squares discriminant analysis of metabolome profiles from oocyte maturation media samples collected before and after 24 h *in vitro* oocyte maturation.

Table S1. Influence of preovulatory follicle maturity on metabolites detected in oocyte maturation media collected before the onset of 24 h in vitro oocyte maturation

Metabolite	P Value	*Fold Change
**1-Methylhistidine**	**2.12E-04**	**1.74**
2-Aminoadipate	1.40E-01	1.08
**2-Dehydro-D-gluconate**	**3.01E-13**	**1.44**
**2-Oxo-4-methylthiobutanoate**	**2.20E-16**	**2.47**
**2,3-Dihydroxybenzoate**	**2.20E-16**	**-1.64**
**3-Methylphenylacetic acid**	**2.20E-16**	**1.34**
3-Methylthiopropionate	9.89E-01	1.00
4-Aminobenzoate	3.14E-01	-1.06
**4-Pyridoxate**	**1.77E-04**	**1.12**
**Acetyllysine**	**2.20E-16**	**1.28**
Acetylphosphate	1.06E-01	-1.23
**Aconitate**	**2.20E-16**	**1.41**
Adenine	6.76E-01	-1.01
AICAR	9.68E-01	1.01
Alanine/Sarcosine	7.15E-01	1.02
Allantoate	1.04E-01	1.22
**Allantoin**	**2.20E-16**	**1.64**
**Alpha-Ketoglutarate**	**2.21E-05**	**1.34**
Aminocaproic acid	7.27E-01	1.01
**Aminoimidazole ribotide**	**3.34E-02**	**-1.78**
AMP/dGMP	7.29E-01	1.06
Arginine	6.57E-01	-1.01
Ascorbate	2.38E-01	1.04
**Asparagine**	**2.20E-16**	**1.60**
Aspartate	2.92E-01	1.02
Biotin	6.53E-01	1.01
Carnitine	5.33E-01	-1.03
**Cholate**	**2.20E-16**	**2.14**
Cholesterol sulfate	1.38E-01	1.15
**Citraconate**	**2.20E-16**	**1.37**
**Citrate/Isocitrate**	**1.24E-07**	**1.29**
**Citrulline**	**2.20E-16**	**1.39**
**Creatine**	**7.03E-05**	**1.26**
**Creatinine**	**1.37E-08**	**1.41**
Cystathionine	2.76E-01	1.56
Cysteate	4.72E-01	-1.06
Cysteine	5.39E-01	-1.03
Cystine	5.46E-01	1.01
Cytidine	6.73E-01	1.09
**Cytosine**	**9.76E-03**	**1.39**
D-Glucarate	7.37E-01	-1.06
D-Gluconate	1.28E-01	1.15
Deoxycytidine	1.40E-01	1.11
**Deoxyinosine**	**3.74E-02**	**-1.58**
FAD	7.74E-01	1.01
Folate	6.10E-01	-1.05
**Glucose phosphate**	**7.00E-03**	**1.73**
Glutamate	3.82E-01	1.02
Glutamine	5.91E-01	1.02
Glutathione disulfide	2.28E-01	1.44
Glycine	8.11E-01	-1.01
**Glycodeoxycholate**	**3.09E-08**	**1.70**
Guanine	8.45E-02	1.28
**Guanosine**	**1.72E-02**	**1.39**
**Histidine**	**1.21E-02**	**1.06**
Homocysteic acid	9.67E-01	1.13
**Homocysteine**	**1.39E-02**	**1.78**
Homoserine/Threonine	6.99E-01	1.01
**Homovanillic acid**	**3.50E-11**	**1.15**
**Hydroxyphenylacetate**	**2.20E-16**	**1.86**
Hydroxyproline	9.18E-01	-1.00
**Hypoxanthine**	**1.38E-02**	**-1.10**
Indole	9.63E-01	-1.00
**Indole-3-carboxylate**	**1.78E-02**	**1.09**
**Indoleacrylate**	**9.40E-03**	**1.46**
Inosine	2.76E-01	1.28
Kynurenic acid	9.02E-02	-1.19
Kynurenine	2.62E-01	1.06
**Lactate**	**2.20E-16**	**-1.70**
Leucine/Isoleucine	7.21E-01	1.01
Lysine	2.45E-01	-1.04
Methionine	5.45E-01	-1.02
Methionine sulfoxide	9.89E-01	1.03
Myo-Inositol	1.45E-01	1.12
**N-Acetyl-beta-alanine**	**2.20E-16**	**1.70**
N-Acetylglucosamine	2.04E-01	1.58
**N-Acetylglucosamine 1-phosphate**	**2.20E-16**	**1.68**
**N-Acetylglutamate**	**3.44E-02**	**1.15**
**N-Acetylornithine**	**2.20E-16**	**1.37**
N-Carbamoyl-L-aspartate	3.21E-01	1.11
**Nicotinate**	**1.23E-02**	**-1.09**
Norepinephrine	6.70E-01	1.01
**Ophthalmic acid**	**2.20E-16**	**1.51**
**Ornithine**	**1.12E-04**	**1.19**
Orotate	3.38E-01	1.08
**Pantothenate**	**2.20E-16**	**1.34**
Phenylalanine	3.76E-01	1.03
**Phenyllactic acid**	**2.20E-16**	**-3.44**
Phosphoenolpyruvate	9.78E-01	1.01
Phosphorylethanolamine	4.64E-01	1.33
Pikatropin	3.19E-01	-1.11
Proline	8.73E-01	1.01
Pyridoxine	3.86E-01	1.03
Pyroglutamic acid	8.87E-01	1.01
**Pyruvate**	**3.40E-02**	**1.05**
Ribose-5-phosphate	8.95E-01	-1.01
S-Adenosyl-L-methioninamine	9.56E-01	1.27
Serine	3.82E-01	1.03
**Taurine**	**7.51E-03**	**1.18**
**Taurodeoxycholate**	**1.01E-03**	**1.41**
**Thymidine**	**8.52E-09**	**-1.72**
Thymine	8.76E-01	1.00
**Tricarballylic acid**	**2.20E-16**	**2.01**
Tryptophan	6.06E-01	1.01
Tyrosine	8.73E-01	-1.00
Uracil	7.03E-01	-1.06
**Uric acid**	**5.98E-08**	**1.20**
**Uridine**	**2.20E-16**	**1.57**
Valine/Betaine	5.23E-01	-1.03
**Xanthine**	**3.22E-05**	**1.12**
Xanthosine 5'-phosphate	5.52E-02	1.78
Xylose	4.30E-01	1.04

* Fold change calculated using greater follicle maturity treatment as reference

Table S2. Influence of preovulatory follicle maturity on metabolites detected in oocyte maturation media collected after 24 h of in vitro oocyte maturation

Metabolite	P Value	*Fold Change
**1-Methyladenosine**	**1.94E-10**	**2.14**
1-Methylhistidine	7.21E-01	1.05
2-Aminoadipate	6.17E-01	1.04
**2-Dehydro-D-gluconate**	**4.84E-14**	**1.43**
2-Hydroxy-2-methylsuccinate	7.57E-02	1.10
2-Hydroxyglutaric acid	6.02E-02	1.10
2-Oxo-4-methylthiobutanoate	5.52E-01	-1.06
2-Oxoisovalerate	7.35E-01	1.02
**2,3-Dihydroxybenzoate**	**1.19E-07**	**-1.34**
**3-Methylphenylacetic acid**	**3.81E-09**	**1.44**
**3-Methylthiopropionate**	**1.20E-03**	**1.17**
**3-Phosphoserine**	**6.28E-03**	**1.37**
**4-Aminobenzoate**	**1.04E-02**	**1.09**
4-Pyridoxate	2.57E-01	1.07
5-Hydroxyindoleacetic acid	4.69E-01	1.09
Acetyllysine	2.72E-01	1.06
Acetylphosphate	3.15E-01	-1.14
**Aconitate**	**6.69E-04**	**1.18**
Adenine	3.82E-01	1.09
**Adenosine**	**1.11E-12**	**-1.89**
AICAR	7.22E-01	-1.11
Alanine/Sarcosine	3.72E-01	1.04
**Allantoate**	**1.59E-04**	**1.29**
**Allantoin**	**8.67E-16**	**1.33**
**Alpha-Ketoglutarate**	**1.56E-04**	**1.15**
Aminocaproic acid	4.61E-01	1.02
**Aminoimidazole ribotide**	**1.15E-02**	**-2.20**
AMP/dGMP	4.74E-01	-1.07
Arginine	1.01E-01	1.04
**Asparagine**	**2.20E-08**	**1.31**
Aspartate	6.76E-01	-1.02
Biotin	8.39E-01	1.01
**Carnitine**	**1.63E-02**	**1.11**
**Cholate**	**2.20E-16**	**2.39**
**Cholesterol sulfate**	**8.40E-11**	**1.53**
**Citraconate**	**6.24E-04**	**1.15**
**Citrate/Isocitrate**	**4.34E-02**	**1.14**
**Citrulline**	**8.71E-14**	**1.29**
**Creatine**	**4.35E-08**	**1.21**
**Creatinine**	**5.27E-06**	**1.23**
Cystathionine	6.25E-01	-1.06
Cysteate	8.57E-01	-1.01
Cysteine	8.52E-01	-1.01
Cystine	7.72E-01	1.01
**Cytidine**	**1.57E-03**	**1.83**
Cytosine	1.44E-01	1.18
D-Glucarate	1.12E-01	1.15
**D-Gluconate**	**5.63E-08**	**1.30**
Deoxycytidine	7.10E-01	1.02
**Deoxyinosine**	**3.84E-03**	**-1.30**
Deoxyuridine	1.45E-01	1.09
FAD	7.74E-01	1.42
Folate	7.06E-02	1.23
Fumarate	6.90E-01	1.03
**Glucose phosphate**	**4.59E-02**	**1.15**
Glutamate	3.74E-01	0.89
**Glutamine**	**1.65E-03**	**1.10**
**Glutathione**	**1.49E-03**	**-1.62**
Glutathione disulfide	2.06E-01	-1.56
Glycerate	6.27E-02	1.11
Glycine	2.90E-01	1.04
**Glycodeoxycholate**	**2.20E-16**	**2.01**
**Guanine**	**2.24E-10**	**1.53**
**Guanosine**	**2.12E-02**	**-1.43**
**Histidine**	**1.83E-04**	**1.11**
**Homocysteic acid**	**4.47E-02**	**1.24**
Homocysteine	2.27E-01	1.20
**Homoserine/Threonine**	**1.55E-02**	**1.09**
Homovanillic acid	6.85E-01	1.03
**Hydroxyphenylacetate**	**2.20E-16**	**2.17**
Hydroxyproline	1.99E-01	1.04
**Hypoxanthine**	**4.77E-04**	**-1.72**
**Indole**	**4.23E-02**	**1.06**
**Indole-3-carboxylate**	**2.66E-04**	**1.25**
**Indoleacrylate**	**5.59E-04**	**1.65**
**Inosine**	**3.66E-02**	**-1.24**
Kynurenic acid	8.55E-01	-1.02
Kynurenine	9.14E-02	1.09
Lactate	3.04E-01	-1.05
Leucine/Isoleucine	4.57E-01	1.02
Lysine	7.20E-01	1.01
Malate	5.38E-01	1.07
Methionine	1.17E-01	1.05
Methionine sulfoxide	5.01E-01	1.05
Myo-Inositol	2.97E-01	1.08
**N-Acetyl-beta-alanine**	**2.20E-16**	**1.72**
**N-Acetylglucosamine**	**1.48E-11**	**2.22**
**N-Acetylglucosamine 1-phosphate**	**5.24E-03**	**1.38**
**N-Acetylglutamate**	**3.10E-03**	**1.15**
**N-Acetylglutamine**	**2.20E-16**	**1.75**
**N-Acetylornithine**	**2.67E-03**	**1.14**
N-Carbamoyl-L-aspartate	2.88E-01	1.07
**Nicotinate**	**1.95E-03**	**-1.09**
**Norepinephrine**	**5.58E-04**	**1.16**
Ophthalmic acid	9.23E-01	-1.01
**Ornithine**	**4.24E-09**	**1.20**
Orotate	1.67E-01	1.11
Pantothenate	7.70E-01	-1.03
**Phenylalanine**	**2.36E-02**	**1.06**
**Phenyllactic acid**	**2.20E-16**	**-2.67**
Phosphorylethanolamine	7.35E-01	1.08
**Pikatropin**	**2.43E-02**	**1.17**
Proline	2.36E-01	1.04
**Pyridoxine**	**4.39E-04**	**1.09**
**Pyroglutamic acid**	**4.68E-04**	**1.09**
Pyruvate	9.01E-01	1.00
Ribose-5-phosphate	4.65E-01	-1.05
S-Adenosyl-L-methioninamine	1.35E-01	1.48
S-Methyl-5'-thioadenosine	6.82E-02	1.14
**Serine**	**6.79E-03**	**1.10**
Succinate/Methylmalonate	3.38E-01	1.06
Taurine	8.09E-01	1.02
**Taurodeoxycholate**	**1.53E-05**	**1.49**
**Thymidine**	**6.80E-03**	**-1.33**
**Thymine**	**2.99E-02**	**1.12**
**Tricarballylic acid**	**2.20E-16**	**2.04**
**Tryptophan**	**2.37E-02**	**1.03**
**Tyrosine**	**4.14E-02**	**1.11**
Uracil	2.94E-01	1.04
**Uric acid**	**2.20E-16**	**1.47**
**Uridine**	**1.59E-07**	**1.26**
Valine/Betaine	2.30E-01	1.04
**Xanthine**	**4.95E-02**	**-1.20**
Xanthosine	7.80E-01	-1.03
**Xanthosine 5'-phosphate**	**8.27E-03**	**1.59**
Xanthurenic acid	4.95E-01	1.11
Xylitol	7.10E-01	1.03
**Xylose**	**2.28E-04**	**1.22**

* Fold change calculated using greater follicle maturity treatment as reference

Table S3. Metabolites in which preovulatory follicle maturity affected change in abundance from onset to completion of 24 h in vitro oocyte maturation

Metabolite	*Change in abundance classification	#Change in abundance fold change difference	P Value
2-Dehydro-D-gluconate	Positive in both treatments	1.83	2.84E-02
3-Methylthiopropionate	Positive in both treatments	1.30	2.21E-02
Allantoate	Positive in both treatments	1.31	4.82E-03
Alpha-Ketoglutarate	Positive in both treatments	1.15	5.12E-04
D-Gluconate	Positive in both treatments	1.42	2.91E-03
Deoxyinosine	Positive in both treatments	-1.30	4.20E-03
Guanine	Positive in both treatments	1.55	3.88E-10
Guanosine	Positive in both treatments	-1.59	6.87E-03
Hypoxanthine	Positive in both treatments	-1.69	1.15E-03
Inosine	Positive in both treatments	-1.44	1.60E-02
Lactate	Positive in both treatments	1.10	4.46E-02
N-Acetylglucosamine	Positive in both treatments	2.15	1.20E-09
Phenylalanine	Positive in both treatments	10.80	1.60E-02
Pyroglutamic acid	Positive in both treatments	1.17	1.89E-02
Uric acid	Positive in both treatments	1.59	< 2.2e-16
Xanthine	Positive in both treatments	-1.69	3.01E-02
Xanthosine 5'-phosphate	Positive in both treatments	1.58	1.97E-02
2-Oxo-4-methylthiobutanoate	Negative in both treatments	8.10	2.50E-07
2,3-Dihydroxybenzoate	Negative in both treatments	-3.09	7.88E-07
Hydroxyphenylacetate	Negative in both treatments	1.77	< 2.2e-16
Indole-3-carboxylate	Negative in both treatments	-3.66	6.94E-03
N-Acetylglucosamine 1-phosphate	Negative in both treatments	1.59	6.11E-06
Phenyllactic acid	Negative in both treatments	-5.98	< 2.2e-16
Carnitine	Negative in lesser and positive in greater follicle maturity	-2.03	1.45E-02
Histidine	Negative in lesser and positive in greater follicle maturity	-25.45	2.73E-02
Indole	Negative in lesser and positive in greater follicle maturity	-2.35	7.89E-03
Methionine	Negative in lesser and positive in greater follicle maturity	-2.80	4.98E-02
Tryptophan	Negative in lesser and positive in greater follicle maturity	-7.53	7.82E-03
Tyrosine	Negative in lesser and positive in greater follicle maturity	-4.98	1.06E-02
Xylose	Negative in lesser and positive in greater follicle maturity	-4.13	4.21E-02

* Metabolite abundance in oocyte maturation media collected after 24 hr in vitro oocyte maturation minus metabolite abundance in oocyte maturation media collected before the onset of in vitro oocyte maturation
# Fold change calculated using greater follicle maturity treatment as reference
